# Effects of 90-Day Feeding of Transgenic Maize BT799 on the Reproductive System in Male Wistar Rats

**DOI:** 10.3390/ijerph121214986

**Published:** 2015-12-02

**Authors:** Qian-ying Guo, Li-xia He, Han Zhu, Jun-li Shang, Ling-yan Zhu, Jun-bo Wang, Yong Li

**Affiliations:** 1 Department of Nutrition and Food Hygiene, School of Public Health, Peking University, Beijing 100191, China; mrLi4j@163.com (Q.G.); helixiapku@163.com (L.H.); zhuhan@bjmu.edu.cn (H.Z.); shangjunli2013@gmail.com (J.S.); zhulingyan2013@163.com (L.Z.); 2 Peking University People’s Hospital, Beijing 100044, China; 3 Beijing Key Laboratory of Toxicological Research and Risk Assessment for Food Safety, Peking University, Beijing 100191, China

**Keywords:** transgenic maize, BT799, *Bacillus thuringiensis*, reproductive toxicity, sperm parameters

## Abstract

BT799 is a genetically modified (GM) maize plant that expresses the Cry1Ac gene from *Bacillus thuringiensis* (Bt). The Cry1Ac gene was introduced into maize line Zhen58 to encode the Bt crystal protein and thus produce insect-resistant maize BT799. Expression of Bt protein *in planta* confers resistance to Lepidopteran pests and corn rootworms. The present study was designed to investigate any potential effects of BT799 on the reproductive system of male rats and evaluate the nutritional value of diets containing BT799 maize grain in a 90-day subchronic rodent feeding study. Male Wistar rats were fed with diets containing BT799 maize flours or made from its near isogenic control (Zhen58) at a concentration of 84.7%, nutritionally equal to the standard AIN-93G diet. Another blank control group of male rats were treated with commercial AIN-93G diet. No significant differences in body weight, hematology and serum chemistry results were observed between rats fed with the diets containing transgenic BT799, Zhen58 and the control in this 13-week feeding study. Results of serum hormone levels, sperm parameters and relative organ/body weights indicated no treatment-related side effects on the reproductive system of male rats. In addition, no diet-related changes were found in necropsy and histopathology examinations. Based on results of the current study, we did not find any differences in the parameters tested in our study of the reproductive system of male rats between BT799 and Zhen58 or the control.

## 1. Introduction

Microbial *Bacillus thuringiensis* (Bt) products with toxicity to Lepidopteran species have been used as insecticides for more than 40 years. Ingestion of select Bt proteins lead to interactions between the Bt protein and receptors in the gut epithelial cells of targeted insect species (but not in mammals) that then result in pore formation and lysis of gut cells, which ultimately causes the insects to die [1,2,3,4]. Genetic engineering technology allows the application of *Bacillus thuringiensis* (Bt) crystalline (Cry) protein genes to crops (e.g., rice, maize, cotton, soy) to protect agricultural plants from insect infection. These crops are usually called transgenic Bt crops [5].

BT799 is a genetically modified Bt-containing maize currently under development in China, produced by insertion of the Cry1Ac gene into the parental maize Zhen58. Expression of the Cry1Ac protein in BT799 provides farmers with an alternative way to decrease yield losses and reduce the use of chemical insecticides. However, there are still concerns about potential unintended effects following the insertion of exogenous gene in crops, especially in staple foods (e.g., rice, maize). In 2009, China’s Ministry of Agriculture released biosecurity certificates for two lines of transgenic Bt crops. This event revealed the commercialization of transgenic crops in China and put this topic on the public agenda [6,7]. In fact, heated disputes about the safety of transgenic crops has not stopped in China since then.

GM crops present challenges concerning food safety assessment because some scientists hold the opinion that unintended effects of transgenic crops due to the insertion of foreign genes are still possible [8,9,10]. The concept of substantial equivalence was proposed as the starting point for safety evaluation of GM crops [11,12]. A GM crop and its non-GM traditional version that has long been consumed in the human diet should be compared with each other in terms of their composition and agronomic performance. Several international organizations also have recommended subchronic rodent feeding studies to investigate any long-term effects of transgenic foods obtained from GM crops [13,14]. A large number of 90-day feeding studies to determine whether unintended adverse effects of GM crops will occur by evaluation of standard toxicology response variables have thus been reported. To date almost no harmful effects have been observed in these studies focusing on GM crops, including maize [15,16,17,18,19], soybean [20,21] and rice [22,23,24,25]. Despite the number of current studies on the safety of GM foods, there are still overwhelming doubts about the immature toxicological assessment procedures used for GM foods. Indeed, some studies have reported obviously harmful effects of GM foods. In the study led by Vecchio pregnant Swiss mice and male litters were fed on a standard diet containing 14% GM soybean. By means of immunoelectron microscopy at 2, 5 or 8 months of age, they focused on Sertoli cells, spermatogonia and spermatocytes. The results showed that GM-fed mice of all ages had larger number of perichromatin granules and lower nuclear pore density [26]. Meanwhile, enlargements of smooth endoplasmic reticulum was also observed in Sertoli cells of GM-fed mice. A three-generation study reported minimal histopathological changes in liver and kidney in F3 female offspring of rats fed a Bt maize [27]. Based on some of studies, Dona and Arvanitoyannis concluded that GM foods appeared to have toxic effect on the reproductive system, and might alter the normal hematological, biochemical, and immunologic parameters [10].

A multifaceted process that involves the combined action of testes, epididymides, accessory sex glands and associated hormones facilitates male reproduction [28]. The hypothalamic-pituitary-testicular axis strictly regulates the whole reproduction process, including the functions of testosterone (T), follicle-stimulating hormone (FSH) and luteinizing hormone (LH). The male reproductive system is usually considered at risk during the fetal development period, during puberty and even over the whole life span due to the sensitivity of the testes and accessory organs. Due to the high rate of cellular proliferation and distinctive differentiation in mammalian testis, molecular and cellular changes can be detected by this very sensitive organ when exposed to toxicants [28,29]. Thus, many scientists and research groups are concerned about possible negative effects that the GM food may exert on the male reproductive system and have conducted a number of studies in order to ensure the safety of GM foods by various methods using different species of animals [30]. Considering sperm maturation needs about 70 days in rats [31,32], a 90-day feeding period would put the male reproductive system at risk if unintended effects happen after exposure to transgenic crops, so studies of that length are often used.

In recent years, concerns about the dietary effects of GM crops on humans and animals have been raised. However, the previous studies cannot respond unequivocally to the public’s doubts or determine unintended effects on the male reproductive system. Considering maize is a widely consumed food in China and a serious debate on the risk assessment of transgenic crops continues, 90-day feeding studies are very necessary to evaluate the male reproductive toxicity of BT799 maize. In this study, we therefore aimed to perform such a study and investigate the toxicological effects of BT799 maize on the growth performance and reproductive parameters of male Wistar rats. The present study was conducted in accordance to the domestic OECD 408 guidelines for Good Laboratory Practices [33].

## 2. Experimental Section

### 2.1. Plant Materials

Transgenic maize (BT799) and its none-transgenic near isoline control maize (Zhen58) were cultivated in the experimental field of China Agricultural University (Beijing, China) and provided by Chinese Center for Disease Control and Prevention (Beijing, China). Seeds of BT799 and Zhen58 were dried, hulled and stored in dry conditions for experimental use after harvesting.

### 2.2. Diet Formulation and Compositional Analysis of Maize

Samples for compositional analysis were randomly selected from both types of maize flour crushed from Zhen 58 and BT 799. Protein, fiber, fat, moisture and ash were determined in accordance with Chinese Standard methods [34,35,36,37,38,39]. Carbohydrate content was calculated using the following expression as described by Han *et al*. [40]:

Carbohydrate % = 100–(% protein + % fat + % ash + % moisture)

Maize flours from BT799 and Zhen58 were respectively added at 84.7% by mass to produce rodent diets. Carbohydrates in the BT799 formula were completely derived from transgenic maize. Casein was added to the feeds in order to make the protein content equivalent to the AIN-93G formula as the amount of protein provided by maize can’t meet the formulation requirements. Other macronutrients in the formulated feeds were added following the same principle as protein when the nutrient content from transgenic maize or non-transgenic maize was lower than in the standard AIN-93G formula. An additional AIN-93G diet was included as negative control. All diets were produced by Hua Fu Kang Feed Co. Ltd. (Beijing, China), following AIN-93G guidelines [41]. Diets formulation details are summarized in Table 1, and the nutritional composition of diets in the different groups is presented in Table 2.

**Table 1:** Composition of diets for rats.

Ingredient (%)	AIN-93G	Zhen58	BT799
Maize	0.00	84.68	84.68
Casein	20.00	11.82	12.54
Cane sugar	10.00	0.00	0.00
Corn starch	52.95	0.00	0.00
Soybean oil	7.00	5.12	4.07
Cellulose	5.00	2.56	2.67
Minerals	3.50	3.50	3.50
Vitamins	1.00	1.00	1.00
Methionine	0.18	0.18	0.18
Choline chloride	0.17	0.17	0.17

**Table 2:** Nutritional composition of diets.

Components (%)	AIN93G	Zhen58	BT799
Crude Protein	20	20	20
Crude Fat	7.0	7.0	7.0
Carbohydrate	62.95	63.99	62.95
Cellulose	5.0	5.0	5.0
Energy, kcal/g	3.77	3.81	3.77

### 2.3. Animals and Housing

A total of 30 male Wistar rats (*n* = 10/group) purchased from Experimental Animal Division of Peking University Health Sciences Center (Beijing, China) were 4 weeks of age at the start of the study and housed in the specific pathogen-free animal facility in a climate controlled room with a 12-h on/off light cycle. This study was approved by the Medical Ethics Committee and performed in accordance with the Guidelines for Animal Experiments of the Peking University Health Science Center. 

### 2.4. Experimental Design

Animals were randomly assigned into three groups according to body weight, fed on the respective diets and provided tap water *ad libitum*. Each group included 10 rats and mean body weight in each group at the beginning of the study did not exceed more than 20% variation. Formulated diets containing either 84.7% BT799 or Zhen58 was provided to the animals in two of the experimental groups. The remaining 10 rats were fed with standard AIN-93G diet as negative control. During the experimental period all animals were clinically observed twice daily for mortality, signs of morbidity or any other noteworthy signs. Blood samples for hematology examination were collected from the tail vein. Samples for serum chemistry and serum hormone evaluation were collected from the abdominal aorta under sodium pentobarbital anesthesia and the animals were fasted overnight to minimize fluctuations of the measured parameters. At the end of the study, animals were sacrificed under sodium pentobarbital anesthesia.

### 2.5. Body Weight Gain and Food Utilization

Body weights were obtained once a week and feed intakes were also measured weekly. Mean feed utilization was determined according to the following calculation:

Mean weekly feed utilization (%) = weekly body weight gain/weekly feed consumption × 100%.

### 2.6. Hematology

Red blood cell count (RBC), white blood corpuscle (WBC), hemoglobin (HGB), hematocrit (HCT), mean corpuscular volume (MCV), mean corpuscular hemoglobin (MCH), mean corpuscular hemoglobin concentration (MCHC), blood platelet count (PLT), red cell distribution width (RDW), were measured by a MEK-7222K cell counter (Nihon Kohden, Tokyo, Japan).

### 2.7. Serum Chemistry

Serum total protein (TP), alanine aminotransferase (ALT), aspartate aminotransferase (AST), alkaline phosphatase (ALP), and concentrations of albumin (ALB), cholesterol (CHOL), creatine (CREA), Glucose (GLUC), blood urea nitrogen (BUN), triglycerides (TG) and lactate dehydrogenase (LDH) were measured by using an Olympus AU400 Clinical Chemistry Analyzer (Olympus, Shizuoka, Japan).

### 2.8. Serum Sex Hormone Levels

Rats in this study were fasted for 12 h and blood samples were collected from the abdominal aorta under anesthesia. Samples were centrifuged at 3000 rpm for 10 min at the temperature of 4 °C to separate serum. Serum level of follicle-stimulating hormone (FSH), luteinizing hormone (LH) and testosterone (T) were measured by a radioimmunoassay (RIA) method using hormone specific kits purchased from the Beijing North Institute of Biological Technology (Beijing, China).

### 2.9. Sperm Motility and Count

Right cauda epididymides of all animals were quickly collected after rats were sacrificed according to Adedara and Farombi [42]. Then epididymides were cut with surgical blades into pieces and incubated in 5 mL pre-warmed DMEM at 37 °C for 10 min to permit the release of sperm. The sperm suspensions for evaluation of sperm motility, head counts and morphology were prepared following the procedure described by Tannenbaum *et al*., [43]. One drop of the sperm suspension was placed on a sterile clean microscope slide. At least 10 microscopic fields were observed at 400× magnification using a phase-contrast microscope (Eclipse E200, Nikon, Tokyo, Japan) and the percentage of motile sperm was recorded [44]. The sperm suspension for sperm head count (0.5 mL) was diluted with physiological saline (9.5 mL), then diluted sperm suspension (10 µL) was transferred to each counting chamber and counted with a hemocytometer. Data were expressed as sperm/mL.

### 2.10. Sperm Morphology

The sperm suspension was smeared out on histological slides and stained with 1% Eosin Y for morphologic examination. A total of two hundred sperm from each animal were used to determine morphological abnormalities [44,45,46].

### 2.11. Organ Weights and Histopathology

A complete gross necropsy was conducted by visual inspection on all the animals following sacrifice. Brain, heart, liver, lung, spleen, kidney, testes weight were immediately examined. Relative weight of each organ (or paired organ weights) was calculated along with final individual body weights. Tissue sections from liver, kidney, intestine, testes and were fixed with 4% buffered formalin and embedded in paraffin, then sectioned to 5 µm and stained with hematoxylin and eosin. Histopathological examination of tissue sections was conducted at the Beijing Key Laboratory of Toxicological Research and Risk Assessment for Food Safety (Beijing, China).

### 2.12. Statistical Analysis

Statistical comparisons were designed to determine whether there were statistically significant differences attributable to consumption of BT799 compared to Zhen58 or the AIN-93G diet. Preliminary tests were applied to test homogeneity for all continuous data (Levenes’s test; and Shapiro-Wilks test). If both of the preliminary tests were not significant, then a one-way ANOVA was conducted. Otherwise, a least squared differences model or a Dennett’s multiple comparison should be conducted to determine whether there were significant differences between groups. Response variables from BT799 and Zhen58 groups were first compared individually with those of the control group, and then the values from the BT799 group were compared with those in the Zhen58 group. A *p*-value of less than 0.05 was considered significant in all analyses.

## 3. Results

### 3.1. Compositional Analysis

Nutritional composition analysis results are listed in Table 3. Contents of macro-nutrients (protein, fat, carbohydrate and dietary fiber) in BT799 maize are similar to that of Zhen58 maize according to the test results and within the normal ranges reported for maize [47,48].

**Table 3:** Nutritional composition of two kinds of maize flour in g/100g.

Nutrients	Zhen58	BT799
Protein	9.71 ± 0.15	8.96 ± 0.17
Fat	2.72 ± 0.49	3.57 ± 0.36
Moisture	9.16 ± 0.22	9.55 ± 0.32
Ash	1.35 ± 0.35	1.31 ± 0.04
Total dietary fiber	3.02 ± 0.22	2.78 ± 0.01
Carbohydrate	73.27 ± 1.21	74.72 ± 2.00

Values are presented as mean ± SD (*n* = 3).

### 3.2. Body Weight Gain and Food Consumption

**Figure 1:**
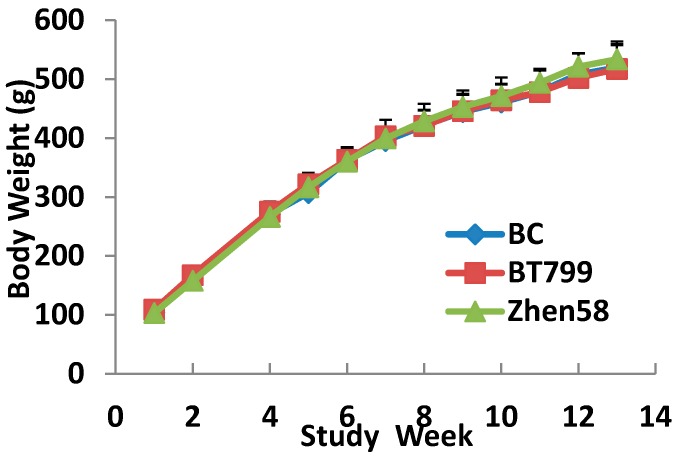
Mean body weights. Values are presented as mean ± SD, analysed by ANOVA, and followed by least-significant difference or Dennett’s multiple comparisons for *post-hoc* comparison between multiple groups.

**Figure 2:**
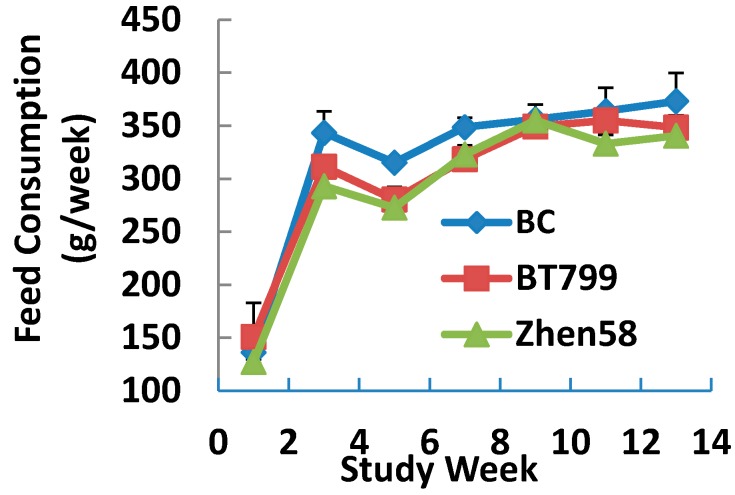
Mean feed consumption. Values are presented as mean ± SD, analysed by ANOVA followed by least-significant difference or Dennett’s multiple comparisons for *post-hoc* comparison between multiple groups.

During the feeding trial, there were no significant differences in weekly body weights (Figure 1) and mean food consumption (Figure 2) between the different groups.

### 3.3. Hematology

No significant differences (*p* > 0.05) observed in the hematology response variables among any of the groups after consuming corresponding formulating diets for 13 weeks (Table 4).

**Table 4:** Hematology and coagulation.

Items	BT799	Zhen58	AIN-93G
WBC (×10^3^/μL)	9.58 ± 2.13	8.62 ± 1.55	8.42 ± 2.47
RBC (×10^6^/μL)	9.19 ± 0.54	8.65 ± 0.45	8.04 ± 0.85
HGB (g/L)	164.13 ± 8.87	167.30 ± 9.01	151.11 ± 23.09
HCT (%)	47.86 ± 2.72	47.22 ± 3.22	42.21 ± 4.14
MCV (fL)	53.06 ± 2.31	54.58 ± 2.28	52.53 ± 2.12
MCH (pg)	18.19 ± 0.63	19.35 ± 1.05	18.79 ± 2.26
MCHC (g/L)	343.12 ± 6.32	355.10 ± 14.89	357.33 ± 36.99
RDW (%)	11.92 ± 0.38	11.97 ± 0.35	12.09 ± 0.27
PLT (×10^3^/μL)	1018.38 ± 28.04	930.10 ± 70.09	1034.00 ± 33.87

Values are presented as mean ± SD, analysed by ANOVA followed by least-significant difference or Dennett’s multiple comparisons for *post-hoc* comparison between multiple groups.

### 3.4. Serum Chemistry

There were no statistically significant changes (*p* > 0.05) observed in any serum chemistry response variables among different groups (Table 5).

**Table 5:** Serum chemistry.

Items	BT799	Zhen58	AIN-93G
TP (g/L)	74.81 ± 2.60	77.39 ± 4.38	71.85 ± 3.27
ALB (g/L)	37.92 ± 1.10	39.14 ± 1.76	36.78 ± 1.06
GLB (g/L)	36.89 ± 1.71	38.25 ± 2.74	35.07 ± 2.45
A/G	1.03 ± 0.03	1.02 ± 0.04	1.05 ± 0.06
ALT (U/L)	48.00 ± 14.41	47.70 ± 12.79	49.30 ± 8.63
ALP (U/L)	157.74 ± 19.28	121.28 ± 36.88	113.19 ± 23.64
BUN (mmol/L)	6.18 ± 0.87	6.82 ± 1.02	7.71 ± 0.86
CHO (mmol/L)	2.19 ± 0.42	2.46 ± 0.50	2.44 ± 0.41
TG (mmol/L)	1.17 ± 0.33	2.11 ± 1.58	1.64 ± 0.37
GLU (mmol/L)	8.16 ± 1.88	6.99 ± 0.74	6.23 ± 0.59
AST (U/L)	158.90 ± 87.22	144.90 ± 55.28	139.80 ± 21.60
LDH (U/L)	1294.56 ± 386.24	1095.81 ± 320.08	1412.06 ± 580.79

Values are presented as mean ± SD, analysed by ANOVA, followed by least-significant difference or Dennett’s multiple comparisons for *post-hoc* comparison between multiple groups.

### 3.5. Serum Sex Hormone

Serum levels of FSH and T were not significantly different among the test groups (Table 6). LH value of BT799 group is higher than that of the Zhen58 group with a significant difference (*p* = 0.013 < 0.05). However, no significant differences were observed between the BT799 group and AIN-93G group.

**Table 6:** Serum hormones concentration.

Items	BT799	Zhen58	AIN-93G
FSH (UI/L)	4.84 ± 0.53	4.97 ± 0.29	5.19 ± 0.31
LH (ng/L)	21.45 ± 1.68 **^#^**	19.53 ± 1.20	20.03 ± 1.45
T (nmol/L)	87.90 ± 5.35	83.36 ± 2.74	92.70 ± 2.59

Values are presented as mean ± SD, analysed by ANONA followed by least-significant difference or Dennett’s multiple comparisons for *post-hoc* comparison between multiple groups. **^#^** *p* < 0.05 compared with Zhen58 group.

### 3.6. Sperm Motility, Sperm Head Counts and Morphology

After the 90-day feeding trial, sperm parameter test results of the three groups exhibited no significant differences (*p* > 0.05), including sperm motility, sperm head counts and morphology of epididymides sperm (Table 7).

**Table 7:** Sperm parameters.

Items	BT799	Zhen58	AIN-93G
Sperm heads (×10^6^/mL)	32.35 ± 1.79	30.50 ± 2.62	31.69 ± 2.33
Sperm motility (%)	87.84 ± 4.00	83.91 ± 6.02	84.06 ± 5.90
Sperm abnormalities (%)	9.44 ± 0.98	9.00 ± 0.93	9.38 ± 0.69

Values are presented as mean ± SD (*n* = 8), analysed by ANOVA followed by least-significant difference or Dennett’s multiple comparisons for *post-hoc* comparison between multiple groups.

### 3.7. Organ Weights and Pathology

There were no statistically significant differences (*p* > 0.05) in the relative organ weights among any test groups (Table 8). 

**Table 8:** Organ/body weight.

Organs	BT799	Zhen58	AIN-93G
Adrenals	0.013 ± 0.004	0.013 ± 0.004	0.010 ± 0.005
Brain	0.328 ± 0.031	0.318 ± 0.025	0.320 ± 0.040
Epididymides	0.220 ± 0.043	0.209 ± 0.028	0.217 ± 0.033
Heart	0.242 ± 0.046	0.250 ± 0.040	0.237 ± 0.027
Kidneys	0.471 ± 0.079	0.506 ± 0.087	0.458 ± 0.059
Liver	2.440 ± 0.391	2.421 ± 0.358	2.155 ± 0.297
Spleen	0.148 ± 0.037	0.147 ± 0.029	0.137 ± 0.022
Testes	0.574 ± 0.096	0.555 ± 0.081	0.579 ± 0.076
Prostate	0.300 ± 0.096	0.290 ± 0.089	0.299 ± 0.092

Values are presented as mean ± SD, analysed by ANONA followed by least-significant difference or Dennett’s multiple comparisons for *post-hoc* comparison between multiple groups.

**Figure 3:**
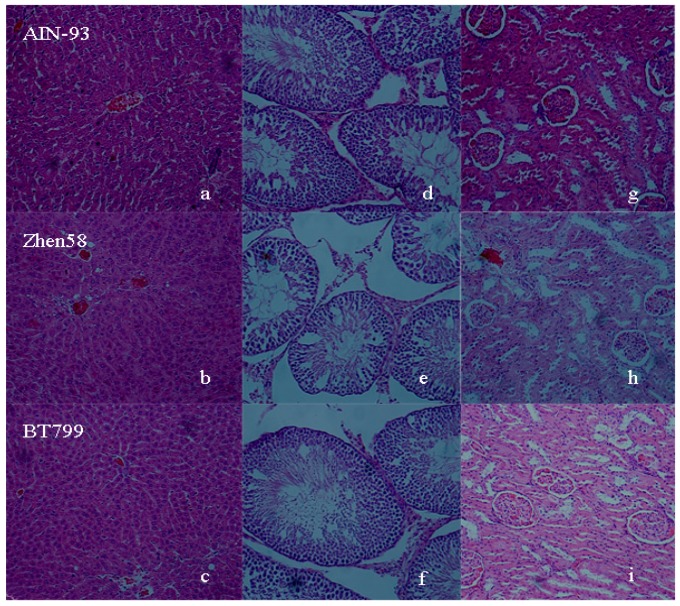
Photomicrographs of liver (**a**–**c**); kidney (**d**–**f**) and testis (**g**–**i**) tissues of male rats stained with H&E × 200. The rows (**a**–**g)**, (**b**–**h**) and (**c**–**i**) were tissues from the AIN-93G control group, Zhen58 group and BT799 group, respectively.

No evidence of altered incidence or severity of pathologic changes was observed in the organs of rats with different diet treatments. Gross and microscopic observations indicated no diet-related changes in the tissues of heart, brain, liver (Figure 3a–c), spleen, kidneys (Figure 3g–i), seminal vesicle, prostate, epididymides and testes (Figure 3d–f) in BT799, Zhen58 and AIN-93G control group.

## 4. Discussion

Guidelines for the safety assessment of genetically modified crops and foods have been developed to assess whether they are “as safe as” conventional non-GM crops [11,12,49,50,51,52]. According to the principle of “substantial equivalence”, the compositional analyses of nutrients in GM crops and comparison between GM crops and conventional non-GM crops are a necessary step for the safety assessment. Rodent feeding studies are also recommended to assess any unintended side effects of transgenic crops [13,14].

In this study, nutritional composition results of transgenic maize and non-transgenic maize indicated that the concentrations of primary nutrients in BT799 were consistent with its near-isogenic maize strain. These results are also consistent with previous studies on GM maize with inserted transgenic genes conducted by other scientists [15,17]. Based on nutrient compositional analysis, transgenic maize flours were added into the diet formulation as the only carbohydrate source and the final BT799 formulation had equal nutritional composition to AIN-93G. A non-transgenic maize diet was formulated under the same equal nutritional principle and it had the same concentration of maize flour (84.7%) in the formulation as BT799 diets. Thus the diet formulations in the different groups had similar energy contents and the basic nutrients demands were also fulfilled.

Most reported subchronic feeding trials have focused on the comparison of nutritional performance between transgenic crops and non-transgenic crops in rodents using diverse responses such as body weight gain, feeding consumption, hematology, serum chemistry. Although a few studies have suggested the consumption of GM crops led to changes in the reproductive parameters of animals, scientific literature concerning the potential effects of GM crops on specific organs or systems are still limited. As a transgenic insect-resistant maize line cultivated in China, BT799 is undisputedly expected to resolve the restricted agricultural production of staple crops. However, it is of great importance to conduct well-designed safety assessments and toxicity studies before a transgenic crop is available on the market. At present, there are no related studies concerning the possible effects on the reproductive system of BT799 maize and the study presented herein definitely fills that gap.

In the present study, sex hormone levels and sperm parameters were given significant attention and examined as the most important indicators of male reproductive function health. The study results showed that a BT799 diet did not affect the nutritional and growth performance of rats. No evident histopathology changes or sperm parameter abnormalities were observed after consumption of BT799 maize. BT799 maize also exhibited similar effects on serum sex hormone levels as the control AIN-93G diet. A higher serum LH level in the BT799 group than the Zhen58 group can be explained as the result of spontaneous alterations (*p* = 0.036 < 0.05). Considering no significant difference was observed between BT799 group and AIN-93G group, the higher LH mean value is not associated with the exposure to transgenic maize.

Transgenic crops, such as rice and maize, are usually consumed by a large number of people worldwide as daily staple foods. Exposure to increased amounts of the expressed protein is quite possible after long-term and multigenerational feeding and this could lead to an adverse effect on the reproductive system if this new protein is a potential toxin [30]. Any long term health impacts after dietary intake of BT799 should be verified before putting this crop on the market. Thus, further safety assessment of BT799 should focus on multigenerational reproductive feeding studies and long-term feeding trials with dietary consumption of the transgenic crop.

## 5. Conclusions

In this study, we observed that the primary nutrient concentrations in BT799 were consistent with those of its near-isogenic maize strain. No significant differences in body weight, hematology and serum chemistry results were observed between rats fed a diet containing transgenic BT799, Zhen58 or the control in this 13-week feeding study. Results of serum hormone levels, sperm parameters and relative organ/body weights indicated no treatment-related side effect on the reproductive system of male rats. In addition, no diet-related changes were found in necropsy and histopathology examinations. Based on results of the current study, we did not find any difference in the parameters tested in our study of the reproductive system of male rats between BT799 and Zhen58 or the control.
